# Corticotropin-Releasing Factor Receptors Modulate Oxytocin Release in the Dorsolateral Bed Nucleus of the Stria Terminalis (BNST) in Male Rats

**DOI:** 10.3389/fnins.2018.00183

**Published:** 2018-03-21

**Authors:** Daisy Martinon, Joanna Dabrowska

**Affiliations:** ^1^Department of Cellular and Molecular Pharmacology, Chicago Medical School, Rosalind Franklin University of Medicine and Science, North Chicago, IL, United States; ^2^Department of Neuroscience, Chicago Medical School, Rosalind Franklin University of Medicine and Science, North Chicago, IL, United States

**Keywords:** oxytocin, CRF, urocortin, BNST, bed nucleus of the stria terminalis, release, microdialysis

## Abstract

The neuropeptide oxytocin (OT) plays an important role in the regulation of social and anxiety-like behavior. Our previous studies have shown that OT neurons send projections from the hypothalamus to the dorsolateral bed nucleus of the stria terminalis (BNST_dl_), a forebrain region critically involved in the modulation of anxiety-like behavior. Importantly, these OT terminals in the BNST_dl_ express presynaptic corticotropin releasing factor (CRF) receptor type 2 (CRFR2). This suggests that CRFR2 might be involved in the modulation of OT release. To test this hypothesis, we measured OT content in microdialysates collected from the BNST_dl_ of freely-moving male Sprague-Dawley rats following the administration of a selective CRFR2 agonist (Urocortin 3) or antagonist (Astressin 2B, As2B). To determine if type 1 CRF receptors (CRFR1) are also involved, we used selective CRFR1 antagonist (NBI35965) as well as CRF, a putative ligand of both CRFR1 and CRFR2. All compounds were delivered directly into the BNST_dl_ via reverse dialysis. OT content in the microdialysates was measured with highly sensitive and selective radioimmunoassay. Blocking CRFR2 with As2B caused an increase in OT content in BNST_dl_ microdialysates, whereas CRFR2 activation by Urocortin 3 did not have an effect. The As2B-induced increase in OT release was blocked by application of the CRFR1 antagonist demonstrating that the effect was dependent on CRFR1 transmission. Interestingly, CRF alone caused a delayed increase in OT content in BNST_dl_ microdialysates, which was dependent on CRF2 but not CRF1 receptors. Our results suggest that members of the CRF peptide family modulate OT release in the BNST_dl_ via a fine-tuned mechanism that involves both CRFR1 and CRFR2. Further exploration of mechanisms by which endogenous OT system is modulated by CRF peptide family is needed to better understand the role of these neuropeptides in the regulation of anxiety and the stress response.

## Introduction

Oxytocin (OT) is a hormone and a neuromodulator produced by neurons in the paraventricular (PVN), supraoptic (SON), and accessory nuclei of the hypothalamus (Sofroniew, 1983; Swanson and Sawchenko, 1983). Central OT neurotransmission is involved in a wide array of social behaviors, including but not limited to, pair bond formation, social recognition, and the onset of maternal behavior (Pedersen et al., 1992; Bosch and Young, 2017). Growing evidence suggests that OT is involved in the regulation of the stress response. For example, OT inhibits hypothalamic-pituitary-adrenal (HPA) axis activity in both male and female rats (Neumann et al., 2000). While the exact mechanisms by which OT regulates stress reactivity are not known, direct interaction between the OT and corticotropin-releasing factor (CRF) systems may substantially impact affective behavior as well as the response to stress. However, little is known about how the CRF-OT systems interact at either in the PVN or extra-hypothalamic sites.

In the 1980's, Bruhn and colleagues showed that intracerebroventricular (ICV) administration of CRF greatly elevates plasma OT levels in rats, without affecting arginine-vasopressin (AVP) secretion (Bruhn et al., 1986), suggesting that central CRF receptor activation could modulate OT secretion. Arima and Aguilera reported that CRFR2 mRNA is co-expressed with OT mRNA in rat SON (Arima and Aguilera, 2000), and we have found similar co-expression of CRFR2, both mRNA and protein, in OT neurons of the rat PVN (Dabrowska et al., 2011). CRF gene expression was significantly enhanced in response to restraint stress in the PVN of OT-deficient mice (Nomura et al., 2003) suggesting that OT secretion can, conversely, impact CRF expression. More recently, activation of the OTR in the PVN was shown to delay transcription of the gene encoding CRF (Jurek et al., 2015), and OT was shown to reduce excitatory synaptic transmission into CRF neurons in the PVN (Jamieson et al., 2017). These studies might provide possible mechanisms for OT regulation of the stress response.

OT neurons are capable of releasing OT from their soma and dendrites in the PVN and SON (Pow and Morris, 1989) as well as from their axons and axon terminals (Ebner et al., 2005). OT fibers originating from the hypothalamus have been found in several brain regions, including the central nucleus of the amygdala (CeA), the lateral septum (LS), and the bed nucleus of the stria terminalis (BNST) (Ebner et al., 2005; Veenema and Neumann, 2008; Dabrowska et al., 2011; Knobloch et al., 2012). Previously, we have shown that OT immunoreactivity in the dorsolateral BNST (BNST_dl_) is restricted to fibers characterized by multiple-beaded varicosities, indicative of possible release sites and axon terminals, which originate, at least partially, from the PVN (Dabrowska et al., 2011). We have also shown that these OT-positive fibers express CRF receptor type 2 (CRFR2). Using electron microscopy we have shown presynaptic localization of CRFR2 on axon terminals that contain dense core vesicles in the BNST_dl_ (Dabrowska et al., 2011). These findings suggest that CRFR2 might play a role in the regulation of OT release in the BNST_dl_.

The effects of CRF-peptide family members are mediated by two receptors: CRFR1 and CRFR2 (Chalmers et al., 1995; Lovenberg et al., 1995), which can be activated by the endogenous peptides, Urocortin (Ucn) 1, 2, and 3 as well as CRF (Hauger et al., 2003a). Ucn3 is the most potent and selective CRFR2 agonist of all, whereas Ucn1 has high affinity for both CRF1 and CRFR2 (Lewis et al., 2001; Suda et al., 2004). CRF has approximately a 17-fold greater affinity toward CRFR1 than CRFR2 (Hauger et al., 2003b); therefore only elevated levels of CRF would be expected to activate both CRFR2 and CRFR1, e.g., following stress. Recently, we have shown that CRFR2 is also present on OT terminals in the nucleus accumbens shell (NaC) of male prairie voles, where it suppresses OT release and is involved in social loss-induced passive coping behavior (Bosch et al., 2016). BNST_dl_ is a key brain area translating stress into sustained anxiety, and has one of the highest densities of oxytocin fibers in the rodent brain (Dabrowska et al., 2011; Knobloch et al., 2012), yet the factors that regulate OT release in the BNST_dl_ are not known. In the current study, we use microdialysis in freely moving rats to demonstrate that OT release in the BNST_dl_ is modulated by members of the CRF peptide family delivered directly into the BNST_dl_ via reverse dialysis. We demonstrate that CRF receptors in the BNST_dl_ play distinct roles in the modulation of OT release such as CRFR2 has inhibitory influence on OT release, whereas intact CRFR1 transmission is necessary for stimulation of OT release in the BNST_dl_.

## Methods

### Animals

Male Sprague-Dawley rats (Envigo, Chicago, IL; 240–300 g at the time of surgery) were housed in groups of 3 with a 12/12 h light-dark cycle and acclimated to the vivarium for at least 7 days before surgery. Protocols for animal experiments in this study were performed in accordance with the guidelines of the National Institute of Health and were approved by the Animal Care and Use Committee at Rosalind Franklin University of Medicine and Science (IACUC protocol # B13-29 and # 17-04). A total of 79 rats were used in the experiments, and nine rats were excluded from the analysis due to probe misplacement or inability to unequivocally confirm placement of the probe. Seventy rats total were included in the analysis.

### Stereotaxic surgery

The two ends of the microdialysis probe were first attached with PE-20 polyethylene tube, followed by flushing and filling of the probe with sterile double distilled water. Standard stereotaxic procedures (Moaddab and Dabrowska, 2017) were used for unilateral implantation of the microdialysis probe. Rats were implanted with probe containing U-shape dialysis membrane (molecular cut-off 18 kDa, Hemophan, Gambro Dialysatoren, Hechingen, Germany), for details see Neumann et al., 1993 into the BNST_dl_ (coordinates from Bregma: AP +0.1 mm, ML +3.4 mm, DV −7.25 mm, 15° coronal angle). Due to transient unavailability of U-shape probes, one group of rats (treated with As2B+CRF, *n* = 6 and ACSF, *n* = 3) was implanted with concentric dialysis membrane: molecular cut-off 50 kDa (Brainlink, Groningen, Netherlands, see Results section for analysis of baseline OT content in BNST_dl_ microdialysates collected with the two types of probes). The length of the microdialysis probes' membrane was 1 mm from the bottom of the shaft to the tip of the membrane. Rats were given an analgesic (5 mg/kg ketoprofen, subcutaneous) prior to the surgery. The surgery was performed with a stereotaxic frame (David Kopf Instruments, USA) using isoflurane anesthesia (E-Z Systems Corporation, Palmer, PA). One group of rats (*n* = 19) was anesthetized during surgery with IP injection of a cocktail dexdormitor (0.25 mg/kg) and ketamine (75 mg/kg) (Henry Schein, Henry Schein Animal Health, Dublin, OH). We have not observed any significant differences in OT levels between rats anesthetized during surgery with the dexdormitor/ketamine cocktail or isoflurane (see Results section for details). Small stainless steel screws were inserted into the frontal and parietal bones to secure the probe to the skull using acrylic cement. At the end of surgery, the outlets of the probe were secured with tape to prevent any damage to the probe until the day of the experiment. Ketoprofen was given again the morning after surgery. Rats were caged individually for 2 days prior to starting the microdialysis experiment. This has been shown to be an optimal timeline as chronic implantation of the microdialysis probe increases the risk of gliosis 3 days after implantation, which significantly reduces absolute and relative recovery of the microdialysis membrane (Hascup et al., 2009).

### Microdialysis

**Figure 1:**
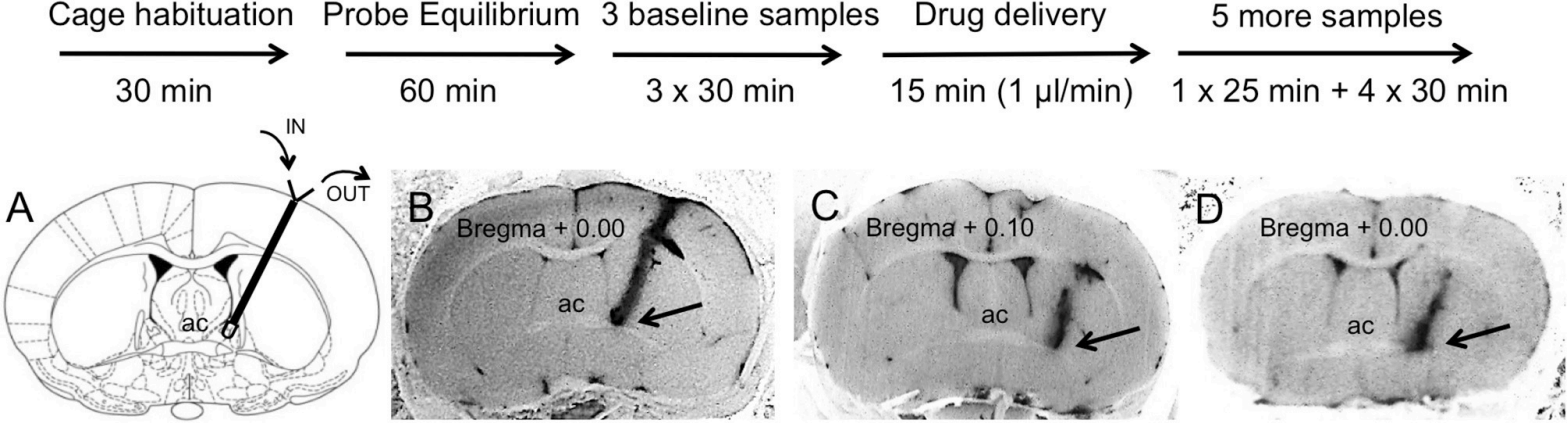
Experimental timeline (upper panel) and representative brain sections with a track of microdialysis probe targeting BNST_dl_ (bottom panel). Upon completion of the microdialysis experiments the probes were perfused with Chicago Sky Blue 6B dye. All extracted brains were sliced and all BNST sections were photographed to confirm proper placement of the probe. **(A)** Only rats with cannula tip located in the BNST_dl_ (Bregma + 0.10 mm to Bregma − 0.36 mm) have been included in the analysis. Modified from Paxinos and Watson (2009). **(B)** An example of confirmed cannula location in the BNST_dl_, which met the following criteria: above the anterior commissure (ac), below the lateral ventricle and medially to the internal capsule as indicated by the arrow (included in the analysis). **(C)** An example of misplaced cannula location with cannula track too lateral to the BNST_dl_ as indicated by the arrow (excluded from the analysis). **(D)** An example of unconfirmed cannula location due to inability to see the tip of the cannula as indicated by the arrow (excluded from the analysis).

Rats were placed individually in Plexiglas chambers (43 × 21 × 31 cm) for 30 min before connecting the probe to the microinjection pump. The rat was gently restrained and the microdialysis probe was connected to a 3 ml syringe mounted on a microdialysis pump (PHD Ultra Pump, Harvard Apparatus) using a 2-channel spiral tubing (CT-20, Eicom, San Diego, CA, internal volume 4 μl) with connecting Joint Teflon (JT-10, Eicom, San Diego, CA, 4 μl) and a 2-way swivel (Eicom, San Diego, CA). Hence, total internal volume of inlet and outlet tubing was 16 μl so that any treatment effects could be observed in the first sample collected after the drug infusion. This microdialysis study was performed in awake, freely moving rats that were provided with food and water for the duration of the experiment. The microdialysis probes were perfused (3.33 μl/min) with sterile Artificial Cerebral Spinal Fluid (ACSF composition: 20 mM NaCl, 3.5 mM KCl, 1.1 mM KH_2_PO_4_, 1.3 mM MgCl_2_, 2.5 mM CaCl_2_, 20 mM glucose, 30 mM NaHCO_3_, 0.4 mM ascorbate, 0.8 mM thiourea, 2 mM Na-pyruvate, pH adjusted to 7.4) for 1 h microdialysis probe equilibration, during which no samples were collected. Following the collection of three 30-min (100 μl each) baseline samples vehicle (ACSF) or vehicle combined with drugs was infused at a flow rate of 1 μl/min for 15 min using 1 ml BD U-100 insulin syringe mounted on a microinjection pump. The slower perfusion rate during this phase of the experiment produced no significant accumulation of OT content in the group perfused only with ACSF (see Results section for details). The flow rate was then restored to 3.33 μl/min for another 25 min and 100 μl samples were collected in each group. Five more, 30 min (100 μl) microdialysate samples were collected while perfusing ACSF only at 3.33 μl/min. All the samples were collected in 1.5-ml low-retention Eppendorf tubes placed on ice and immediately frozen on dry ice after collection and then stored at −80°C. Rats were individually housed for the duration of the microdialysis experiment. Each rat was used only once in the microdialysis experiment and received only one treatment. Experimental timeline is shown on Figure 1.

### Pharmacological manipulations

CRFR2 agonist, Urocortin 3 (mouse) trifluroacetate (Ucn3) and CRF acetate salt (human, rat, synthetic) were purchased from Bachem (catalog number H-5828 and H-2435, respectively, Torrance, CA). CRFR2 antagonist, Astressin-2B (As2B) and CRFR1 antagonist (NBI 35965) were purchased from Tocris (2391 and 3100, respectively, Minneapolis, MN). Drugs were infused at a concentration of 10 μM, a dose that was proven effective in previous microdialysis studies (Ji and Neugebauer, 2007; Wang et al., 2007). Additionally, As2B dose was established based on its efficacy demonstrated on CeA slices during electrophysiological recordings (Pollandt et al., 2006). Drug concentration needed in the microdialysis probe (10 μM) is considered ~100 times higher than effective concentration used in the tissue slice (100 nM), due to the concentration gradient across the dialysis membrane and diffusion in the tissue (Li and Neugebauer, 2004a,b).

Microdialysates were collected in 30-min intervals, with 3-baseline samples followed by pharmacological challenge: Ucn3 (*n* = 7), As2B (*n* = 14), CRF (*n* = 9), As2B + CRF (*n* = 7), As2B + NBI 35965 (*n* = 6), CRF + NBI 35965 (*n* = 8), and ACSF (*n* = 19). All the compounds (CRF, As2B, Ucn3, NBI 35965) have molecular weights below 5 kDa (respectively 4757.52, 4041.69, 4172.97, and 437.79 g/mol); and therefore could be delivered via retrodialysis with microdialysis probes with cut-off of 18 kDa (U-shape) and 50 kDa (concentric). On any experimental day, treatment groups were counterbalanced such as at least one vehicle-infused rat was accompanied by at least two more rats from respective treatment groups (e.g., As2B alone, and As2B + CRF). A pH test strip was used to determine if there was any change in pH after adding the drug to the ACSF.

### Probe placement

Following microdialysis, the rats were deeply anesthetized with isoflurane, the probes were perfused with Chicago Sky Blue 6B dye (Alfa Aesar, Ward Hill, MA) as a 2% solution in 0.9% saline, and the animals were euthanized by decapitation. All extracted brains were frozen, then sliced (50 μm) and photographed to confirm proper placement of the probe (Figure 1). Microdialysates from experimental subjects with correctly placed probes were analyzed for OT content. Proper probe placement met the following criteria: cannula tip located in the BNST_dl_ (Bregma + 0.10 mm to Bregma − 0.36 mm), above the anterior commissure, below the lateral ventricle and medially to the internal capsule (as indicated in Figure 1A). Due to a small number of rats confirmed as negative controls based on unequivocal cannula misplacement (Figure 1), microdialysates from these rats were not analyzed (*n* = 4).

### Radioimmunoassay for OT

The frozen dialysates samples were evaporated to dryness in a vacuum concentrator (Jouan RC10.10, Thermo Fisher Scientific) with a freeze dry system (FreeZone 6, LABCONCO). All evaporated microdialysates were treated identically. The content of OT in each dialysate was quantified with a highly sensitive (0.1 pg OT/100 μl sample) and selective radioimmunoassay (RIA, minimal affinity for arginine-vasopressin, RIAgnosis, Munich, Germany), as described previously (Neumann et al., 1993; Ross et al., 2009; Bosch et al., 2016). Cross-reactivity of the polyclonal antiserum with arginine–vasopressin and other related peptides was <0.7%. Intra- and inter-assay coefficients of variation were <8 and <11%, respectively. Due to the high total number of microdialysis samples (560), microdialysates were analyzed during four RIAs and each assay contained balanced number of samples from different treatment groups.

### Statistics

Data are presented as mean ± standard error of mean (SEM) of OT content in BNST_dl_ microdialysates expressed as pg per 100 μl sample (Table 1). Data sets were first analyzed for normal distribution using D'Agostino & Pearson omnibus normality test. Data demonstrated normal distribution (*P* > 0.05), and was analyzed using parametric tests. Results were first analyzed by within group one-way repeated measures analysis of variance (ANOVA) for each treatment group. Where the F-ratio was significant, all-pairwise *post-hoc* comparisons were made using Bonferroni's tests by comparing the mean of each time point (post-treatment) with the mean of the three baseline samples (pre-treatment). F ratio (DFn, DFd) referred to two degrees of freedom, where DFn = a-1, whereas DFd = N-a. “A” was the number of groups and “N” was the total number of subjects in all groups. Since the sphericity (equal variability of differences) was not assumed in one-way repeated measures ANOVA, the Geisser-Greenhouse correction resulted in smaller degrees of freedom, which were not integers.

**Table 1:** CRFR manipulation affects OT content in BNST_dl_ microdialysates.

**Treatment (*n* per group) OT pg/100 μl MEAN ± SEM**	**Baseline 1**	**Baseline 2**	**Baseline 3**	**30 min**	**60 min**	**90 min**	**120 min**	**150 min**
ACSF (*n* = 19)	1.07 ± 0.05	1.03 ± 0.05	1.04 ± 0.06	1.04 ± 0.11	0.94 ± 0.04	0.93 ± 0.07	0.92 ± 0.06	0.87 ± 0.07
As2B (*n* = 14)	1.31 ± 0.16	1.13 ± 0.18	1.36 ± 0.25	1.79 ± 0.27	1.20 ± 0.16	1.44 ± 0.18	1.29 ± 0.21	1.29 ± 0.17
CRF (*n* = 9)	0.96 ± 0.17	1.08 ± 0.20	1.02 ± 0.14	1.12 ± 0.26	1.06 ± 0.19	1.30 ± 0.23	1.01 ± 0.20	1.07 ± 0.23
Ucn3 (*n* = 7)	1.32 ± 0.10	1.68 ± 0.20	1.30 ± 0.11	1.73 ± 0.16	1.52 ± 0.11	1.57 ± 0.09	1.25 ± 0.06	1.25 ± 0.10
As2B+CRF (*n* = 7)	1.26 ± 0.09	1.27 ± 0.13	1.23 ± 0.12	1.11 ± 0.09	1.07 ± 0.06	1.01 ± 0.08	1.03 ± 0.09	1.08 ± 0.11
CRF+NBI 35965 (*n* = 8)	0.88 ± 0.07	0.91 ± 0.10	1.04 ± 0.06	0.97 ± 0.12	1.06 ± 0.11	1.07 ± 0.08	0.98 ± 0.08	1.04 ± 0.12
As2B+NBI 35965 (*n* = 6)	0.81 ± 0.09	0.98 ± 0.07	1.02 ± 0.10	0.88 ± 0.08	0.93 ± 0.03	0.87 ± 0.07	0.92 ± 0.12	0.91 ± 0.12

*OT content in BNST_dl_ microdialysates is expressed as MEAN ± SEM of OT (pg) in 100 μl microdialysis samples for each treatment group. Microdialysates were collected in 30 min intervals at baseline, and 30, 60, 90, 120, and 150 min after a drug delivery. Three baseline samples were collected in 30 min intervals before a drug delivery (ACSF, Artificial Cerebral Spinal Fluid; AS2B, Astressin 2B; CRF, Corticotropin-releasing factor; Ucn3, Urocortin 3; NBI 35965, CRFR1 antagonist). Underlined: Main treatment effect was observed in rats perfused with As2B, and post-hoc analysis showed significantly increased OT content in the first sample collected after drug infusion (30 min, P < 0.05) in comparison to pre-treatment OT content (Bonferroni's multiple comparisons)*.

For between treatment groups' analysis, data are presented as percentage changes from baseline ± SEM. Here, OT content in microdialysates for each rat was expressed as percentage change from its own baseline values (mean of three baseline samples, 100%) for each time point measured. Here, results were analyzed by two-way repeated measures ANOVA with the factors TIME (measured after a drug infusion) and TREATMENT (ACSF, Ucn3, As2B, CRF, As2B + CRF, As2B + NBI 35965, CRF + NBI 35965). Where the F-ratio was significant, all-pairwise *post-hoc* comparisons were made using Bonferroni's tests.

OT content in two subsequent microdialysates (before and after reduction of the perfusion rate) in control rats perfused with ACSF only was compared using a paired *t*-test. Baseline OT contents in BNST_dl_ microdialysates collected in rats implanted with U-shape vs. Brainlink probes were compared using an unpaired *t*-test. The effect of type of anesthesia used during surgery on OT content in BNST_dl_ microdialysates was also analyzed using an unpaired *t*-test. The statistical analyses were completed using GraphPad Prism version 6.0 (GraphPad Software Inc., San Diego, CA). *P* < 0.05 was considered significant.

## Results

### OT content in BNST_dl_ microdialysates following CRFR modulation—within group analysis

Baseline OT content in the BNST_dl_ from all rats had an average ± SEM of 1.18 ± 0.05 pg. OT content (pg/100 μl) in BNST_dl_ microdialysates, including three baseline samples, is shown in Table 1 for all treatment groups. First, to determine if OT content in baseline BNST_dl_ microdialysates was stable before any treatment was introduced; we have compared oxytocin content between three baseline microdialysates in all rats for each treatment group with one-way repeated measures ANOVA. We found that OT content did not differ between baseline microdialysates in ACSF [*F*_(1.823, 31.00)_ = 0.0536, *P* = 0.93], As2B [*F*_(1.229, 11.06)_ = 0.0684, *P* = 0.85], Ucn3 [*F*_(1.450, 7.249)_ = 1.895, *P* = 0.22], CRF [*F*_(1.844, 14.75)_ = 0.7142, *P* = 0.49], CRF + As2B [*F*_(1.947, 11.68)_ = 0.0609, *P* = 0.94], As2B + NBI 35965 [*F*_(1.489, 7.444)_ = 1.482, *P* = 0.28], and CRF + NBI 35965-treated groups [*F*_(1.185, 8.296)_ = 3.541, *P* = 0.09].

**Figure 2:**
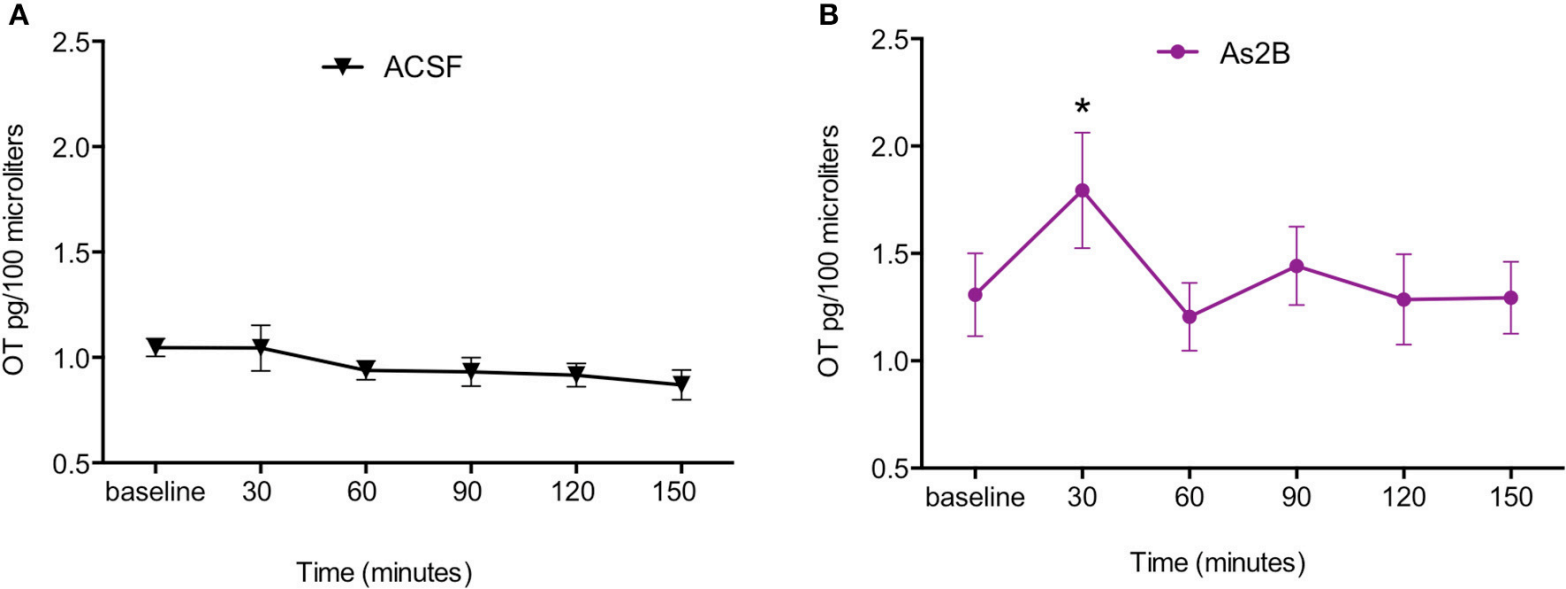
CRFR2 blockade increases OT content in BNST_dl_ microdialysates. OT content in BNST_dl_ microdialysates is expressed as MEAN ± SEM of OT (pg) in 100 μl microdialysis samples for ACSF **(A)** and As2B **(B)** treated groups. Baseline samples were collected in 30 min intervals before a drug delivery. **(A)** Perfusion of ACSF alone did not affect OT content in BNST_dl_ microdialysates. **(B)** There was a significant main treatment effect in rats perfused with As2B (one-way ANOVA), and *post-hoc* analysis showed significantly increased OT content in the first sample collected after drug infusion (30 min, ^*^P < 0.05) in comparison to pre-treatment OT content (Bonferroni's multiple comparisons).

We then compared OT content in BNST_dl_ microdialysates within each treatment group with one-way repeated measures ANOVA. No significant effect of treatment was observed in rats perfused with ACSF only [*F*_(2.972, 53.50)_ = 1.477, *P* = 0.23, Figure 2A]. This suggests that slowing down perfusion rate during drug delivery did not affect OT content in BNST_dl_ microdialysates. There was a significant main treatment effect in rats perfused with As2B [*F*_(2.260, 29.38)_ = 6.921, *P* = 0.0025]. *Post-hoc* analysis has shown significantly increased OT content in the first sample collected after drug infusion (30 min, *P* < 0.05, Figure 2B) in comparison to pre-treatment OT content (Bonferroni's multiple comparisons). Although there was a significant main effect of Ucn3 treatment [*F*_(2.383, 14.30)_ = 3.561, *P* = 0.0493], Bonferroni's multiple comparisons test did not show any differences between OT content at baseline and post-treatment microdialysates at any given time point. Application of CRF alone did not have any main treatment effect on OT content in microdialysates [*F*_(3.048, 24.39)_ = 2.051, *P* = 0.13]. Combination of CRF and As2B also did not affect OT content in BNST_dl_ microdialysates [*F*_(1.948, 11.69)_ = 2.082, *P* = 0.17]. Similarly, co-application of As2B with CRFR1 antagonist, NBI 35965, did not influence OT content in microdialysates [*F*_(2.700, 13.50)_ = 0.1188, *P* = 0.93]. Finally, treatment with CRF and NBI 35965 did not have any main effects on OT release [*F*_(3.123, 21.86)_ = 0.5968, *P* = 0.63], Table 1.

To further exclude the possibility that the slower perfusion rate during drugs delivery (1 μl/min for 15 min) might have altered OT content in BNST_dl_ microdialysates (independently of treatment), we have performed additional analysis in control rats (perfused with ACSF only). Here, we have compared OT levels in baseline microdialysates (mean of three baseline values) with OT levels in microdialysates collected during slower perfusion rate. The analysis showed no difference in OT content between the microdialysates collected before and after slowing down of perfusion rate (*P* = 0.98, *n* = 19, paired *t*-test).

### Percentage change of OT content in BNST_dl_ microdialysates following CRFR modulation—between groups analysis

OT content was expressed as a percent change from baseline based on the average of three baseline values (100%) for each rat. With *post-hoc* analysis, all treatment groups (including ACSF) were compared with each other in their respective time-points after a drug delivery. Comparing percentage changes in OT content in BNST_dl_ microdialysates across all (7) treatment groups with two-way repeated measures ANOVA revealed a significant main effect of TREATMENT [*F*_(6, 63)_ = 3.315, *P* = 0.0067], and a significant effect of TIME [*F*_(5, 315)_ = 2.417, *P* = 0.0359], as well as significant interaction between TIME and TREATMENT [*F*_(30, 315)_ = 1.549, *P* = 0.0367].

**Figure 3:**
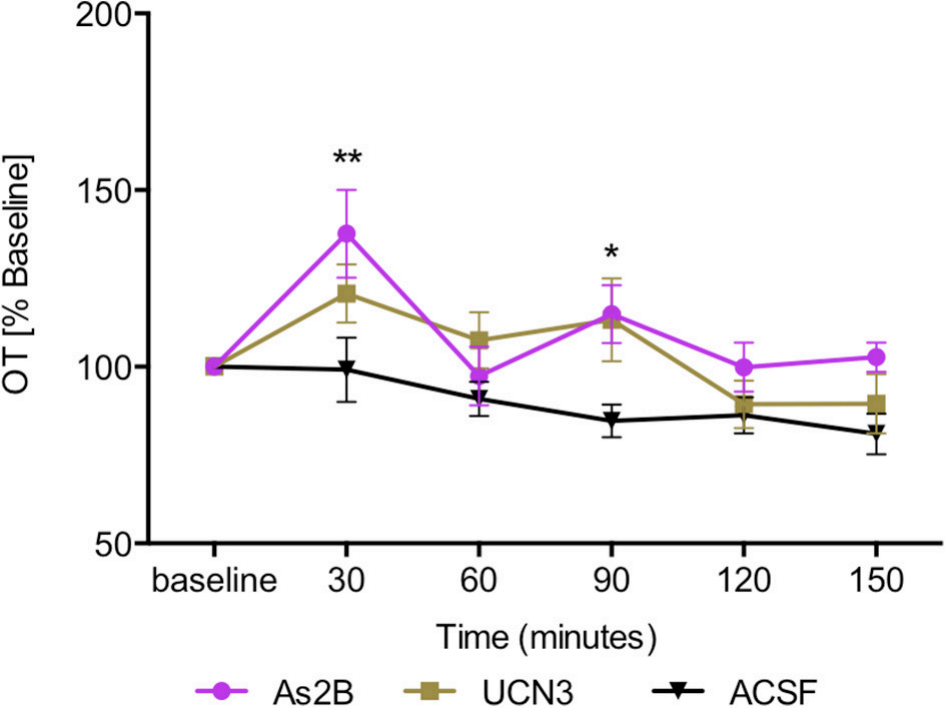
Manipulation of CRFR2 affects OT content in BNST_dl_ microdialysates. OT content in the microdialysates collected at 30 min intervals is expressed as percent change from the baseline. Data is expressed as mean ± SEM. Although CRFR2 activation by Ucn3 did not significantly affect OT release (*n* = 7, olive, closed square), an increase in OT release at 30 and 90 min was induced by intra-BNST_dl_ application of the CRFR2 antagonist, As2B (*n* = 14, purple, closed circle) in comparison to vehicle (ACSF) group (*n* = 19, black, closed triangle, *P* < 0.01). ^**^P < 0.01, ^*^P < 0.05, two-way ANOVA with repeated measures; Bonferroni's multiple comparisons test.

**Figure 4:**
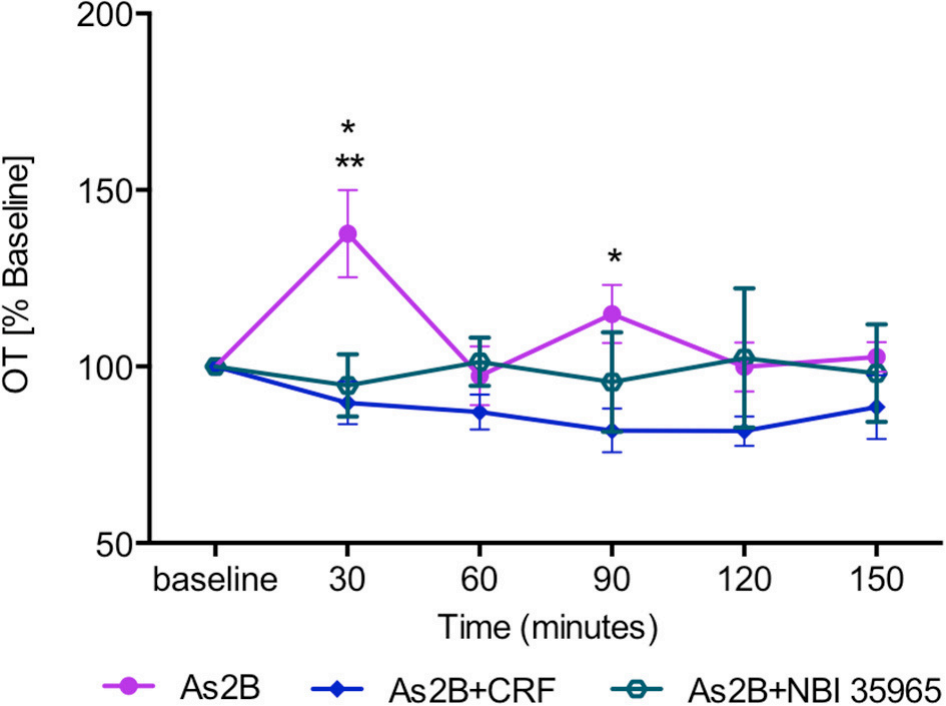
The effect of CRFR2 antagonist (As2B) on OT content in BNST_dl_ microdialysates is modulated by CRFR1 manipulation. The effect of As2B on OT release at 30 min was occluded by intra-BNST_dl_ application of CRFR1 antagonist NBI 35965 (*n* = 6, teal, open circle, *P* < 0.05) as well as application of CRF (*n* = 7, blue diamond, *P* < 0.01). The delayed stimulatory effect of As2B at 90 min was not affected by CRF or NBI 35965 application (*P* > 0.05). ^**^P < 0.01, ^*^P < 0.05, two-way ANOVA with repeated measures.

*Post-hoc* analysis with Bonferroni's multiple comparison test revealed that infusion of CRFR2 agonist (Ucn3) did not affect OT release in the BNST_dl_ at any time point in comparison to any other treatment group (*P* > 0.05, Figure 3). In contrast, infusion of CRFR2 antagonist alone (As2B) caused a significant increase in OT release measured at 30 min (first sample collected after the drug delivery) in comparison to the vehicle (ACSF) (*P* < 0.01, Figure 3) and CRF-treated groups (*P* < 0.05). The effect of As2B was significantly reduced when As2B was co-infused with CRF (*P* < 0.01 in comparison to As2B alone). The effect of As2B was also significantly reduced when As2B was co-infused with CRFR1 antagonist, NBI 35965 (*P* < 0.05 in comparison to As2B alone, Figure 4). In addition, As2B produced a delayed increase in OT content in BNST_dl_ microdialysate 90 min after the drug infusion (*P* < 0.05 in comparison to ACSF-treated group), which was not affected by local application of CRFR1 antagonist, NBI 35965, or CRF (*P* > 0.05) (Figure 4).

**Figure 5:**
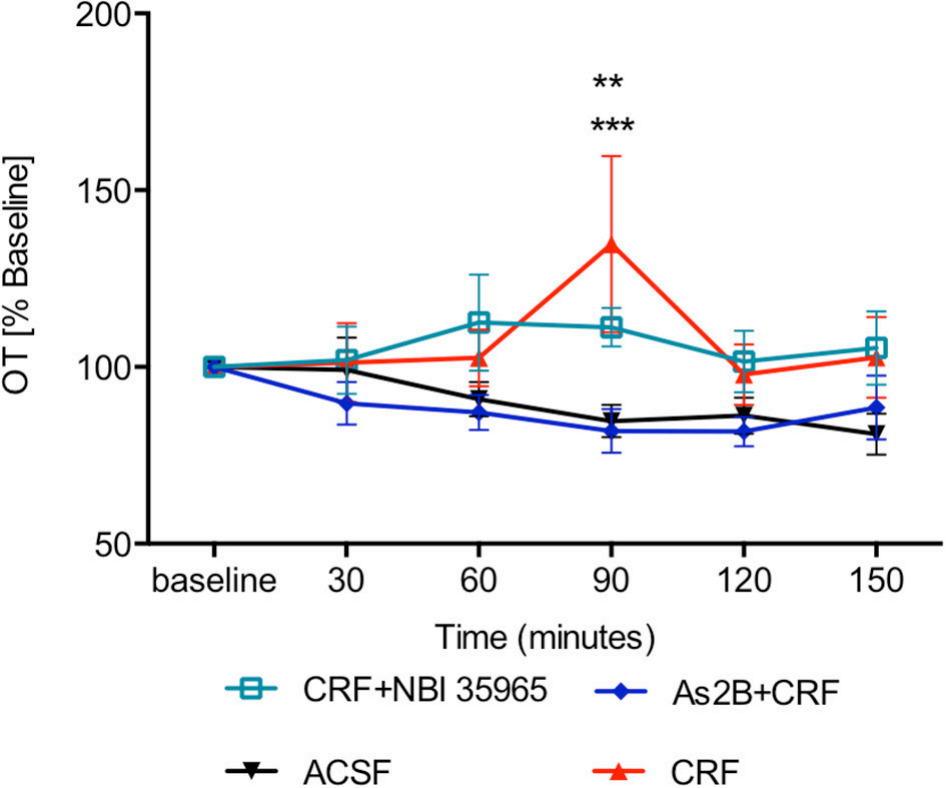
OT content in BNST_dl_ microdialysates is modulated by CRF. CRF caused a significant increase in OT release at 90 min (*n* = 9, red triangle, *P* < 0.001 in comparison to ACSF group). CRFR1 blockade by NBI 35965 did not significantly affect the effect of CRF on OT release (*n* = 8, cyan, open square, *P* > 0.05). However, blocking CRFR2 with As2B abolished the effect of CRF on OT release (*n* = 7, blue diamond, *P* < 0.01). ^***^P < 0.001, ^**^P < 0.01 two-way ANOVA with repeated measures.

Although application of CRF alone did not have any effect immediately after the drug infusion (30 min, *P* > 0.05 in comparison to ACSF-treated rats), it induced a significant delayed increase in OT content in BNST_dl_ microdialysate at 90 min (Figure 5), in comparison to the control (ACSF) group (*P* < 0.001). Interestingly, the delayed stimulatory effect of CRF on OT release was blocked by co-infusion with As2B (*P* < 0.01) (Figure 5), but not CRFR1 antagonist, NBI 35965 (*P* > 0.05) (Figure 5). This suggests that the delayed stimulatory effect of CRF on OT release in the BNST_dl_ is mediated by local CRF2, but not CRF1 receptors.

None of the treatments affected OT content in BNST_dl_ microdialysates at 60, 120, and 150-min after the drug infusions (*P* > 0.05, Figures 3–5).

### OT content in BNST_dl_ microdialysates—technical considerations

#### Microdialysis membrane cut-off and OT content in BNST_dl_ microdialysates

We have compared baseline OT levels in rats implanted with Brainlink concentric probes (*n* = 9) and rats implanted with U-shape probes (*n* = 61) and found a statistical difference (Brainlink 1.25 pg ± 0.05, and U-shape 1.01 pg ± 0.04, *P* = 0.0013, unpaired *t*-test). This is most likely due to a higher cut-off of microdialysis membrane in Brainlink probes in comparison to U-shape probes (50 vs. 18 kDa). We used percentage changes of OT content (calculated as a change from the baseline value for each rat) that allowed comparisons between treatment groups using two-way repeated measures ANOVA, hence higher absolute OT content in BNST_dl_ microdialysates collected with Brainlink probes should not affect the results. However, to ensure that our major findings are not compromised by the data collected from rats implanted with Brainlink probes (As2B+CRF *n* = 6 and ACSF *n* = 3), we have removed the subjects from data analysis. Notably, the exclusion did not affect our main findings. Here, two-way repeated measures ANOVA of all six treatment groups (ACSF, Ucn3, As2B, CRF, As2B + NBI 35965, CRF + NBI 35965) revealed a significant main effect of TREATMENT [*F*_(4, 47)_ = 3.630, *P* = 0.0117], and a significant main effect of TIME [*F*_(5, 235)_ = 2.960, *P* = 0.0130], but not a significant interaction between TIME and TREATMENT [*F*_(20, 235)_ = 1.589, *P* = 0.0562]. *Post-hoc* analysis with Bonferroni's multiple comparison test revealed that infusion of Ucn3 did not affect OT release in the BNST_dl_ (*P* > 0.05), whereas infusion of As2B caused a significant increase in OT release measured at 30 min in comparison to vehicle (ACSF). (*P* < 0.01) and CRF-treated group (*P* < 0.05). The effect of As2B was significantly reduced when As2B was co-infused with CRFR1 antagonist (*P* < 0.05). In addition, As2B also produced a delayed increase in OT content in BNST_dl_ microdialysate at 90 min (*P* < 0.05 in comparison to the ACSF-treated group), which was not affected by local application of the CRFR1 antagonist, NBI 35965 (*P* < 0.05). Although application of CRF alone did not have any effect immediately after the drug infusion (30 min, *P* > 0.05 in comparison to ACSF-treated rats), CRF induced a delayed significant increase in OT content in BNST_dl_ microdialysate at 90 min in comparison to the control (ACSF) group (*P* < 0.001). The delayed stimulatory effect of CRF on OT release was not affected by co-infusion of CRFR1 antagonist (*P* > 0.05).

#### Type of anesthesia during surgery and OT content in BNST_dl_ microdialysates

Here, we have compared baseline OT content in BNST_dl_ microdialysates between rats anesthetized during surgery with isoflurane (1.13 pg ± 0.06, *n* = 51) or ketamine/dexdormitor mixture (1.31 pg ± 0.06, *n* = 19) and found no statistical difference between the two groups of animals (*P* = 0.15, unpaired *t*-test). To determine whether type of anesthesia during surgery could interfere with treatment and therefore affect OT content in BNST_dl_ microdialysates, we have performed two-way ANOVA for the As2B-treated group with factors TREATMENT (pre-drug, post-drug) and ANESTHESIA (isoflurane or cocktail of ketamine/dexdormitor). We chose the As2B group because of the significant effect of As2B on OT content in BNST_dl_ microdialysates (see results above) and because this group contained equal numbers of rats anesthetized with isoflurane (*n* = 7) and rats anesthetized with ketamine/dexdormitor (*n* = 7) during surgery. We have noted a significant effect of As2B TREATMENT [*F*_(1, 12)_ = 12.09, *P* = 0.0046], but there was no significant effect of ANETHESIA [*F*_(1, 12)_ = 0.2673, *P* = 0.61], nor interaction between the TREATMENT and ANETHESIA [*F*_(1, 12)_ = 0.8593, *P* = 0.37].

#### Peptide retrodialysis and OT content in the microdialysates—*ex vivo* experiment

To determine whether peptides used in the treatment groups (CRF, Ucn3, and As2B) could potentially interfere with the OT antibody used in the RIA, we have performed an *ex vivo* experiment in which U-shape microdialysis probes were perfused with ACSF only (*n* = 6 samples collected), or with ACSF containing CRF (*n* = 4), or Ucn3 (*n* = 4), or As2B (*n* = 4) at the concentration and the flow rate as above. The samples were collected into Eppendorf tubes as above and sent for RIA analysis together with the samples collected from *in vivo* microdialysis. Samples obtained from CRF and As2B perfusion were not significantly different from samples perfused with ACSF only (*P* = 0.58 and *P* = 0.13, respectively, unpaired *t*-test). However, samples perfused with Ucn3 had significantly higher levels of OT than samples perfused with ACSF only (*P* = 0.0488, unpaired *t*-test), suggesting that Ucn3 might potentially interact with the RIA, leading to false positive results. To ensure that this has not interfered with our major findings, we have excluded the Ucn3-treated group from the analysis. Notably, exclusion of the Ucn3 group did not affect our major findings in any significant way. Here, two-way repeated measures ANOVA of six treatment groups (ACSF, As2B, CRF, As2B + CRF, As2B + NBI 35965, CRF + NBI 35965) revealed a significant main effect of TREATMENT [*F*_(4, 50)_ = 4.468, *P* = 0.0036], no significant effect of TIME [*F*_(5, 250)_ = 1.346, *P* = 0.24], but a significant interaction between TIME and TREATMENT [*F*_(20, 250)_ = 1.776, *P* = 0.0236].

*Post-hoc* analysis with Bonferroni's multiple comparison test confirmed that infusion of As2B caused a significant increase in OT release measured at 30 min in comparison to the vehicle (ACSF) (*P* < 0.01) and CRF-treated groups (*P* < 0.05). The effect of As2B was significantly reduced when As2B was co-infused with the CRFR1 antagonist (*P* < 0.05), or CRF (*P* < 0.01). In addition, As2B also produced a delayed increase in OT content in BNST_dl_ microdialysate at 90 min (*P* < 0.05 in comparison to ACSF-treated group), which was not affected by application of CRFR1 antagonist (*P* > 0.05). Although application of CRF alone did not have any effect immediately after the drug infusion (30 min, *P* > 0.05 in comparison to ACSF-treated rats), it induced a delayed significant increase in OT content in BNST_dl_ microdialysate at 90 min in comparison to the control (ACSF) group (*P* < 0.001), which was significantly reduced by infusion of As2B (*P* < 0.01), but not CRFR1 antagonist (*P* > 0.05).

## Discussion

Using *in vivo* microdialysis in freely-moving male rats we demonstrate for the first time that OT release in the BNST_dl_ is modulated by members of the CRF-peptide family and that CRFR1 and CRFR2 play distinct roles in this modulation. Our results show that blockade of CRFR2 with As2B caused an increase in OT content in BNST_dl_ microdialysates. Analysis of percentage change of OT content between the treatment groups show that the stimulatory effect of AS2B depends on intact CRFR1 transmission, because the effect of As2B was abolished when CRFR1 were blocked at the same time. In addition, although CRF alone had no immediate effect on OT release, it had a delayed effect on OT content in BNST_dl_ microdialysates, which was blocked by intra-BNST_dl_ application of CRFR2 but not CRFR1 antagonist.

In contrast to widely expressed CRFR1 in rat brain, expression patterns of CRFR2 are more distinct. For example, although CRFR2 mRNA has been found in many brain regions including posterior BNST, its mRNA expression is sparse in the BNST_dl_ or CeA (Chalmers et al., 1995; Van Pett et al., 2000). However, like CRFR1, which has been found at both pre- (Nie et al., 2009) and post-synaptic sites (Fu and Neugebauer, 2008), pre-synaptic CRFR2-mediated modulation of neuronal excitability has been demonstrated in the CeA (Fu and Neugebauer, 2008), LS and nucleus of the solitary tract (Lawrence et al., 2002). Therefore, the previous and current findings indicate that in the BNST_dl_ CRFR2 localized on OT fibers modulate OT release via a pre-synaptic mechanism.

Our finding that selective activation of CRFR2 by Ucn3 did not have any effect on OT content in BNST_dl_ microdialysates appears in contrast to previously reported findings in NaC in male prairie voles, where Ucn3 reduced release of OT. However, it should be noted that the decrease observed in the latter study was statistically significant when comparing OT content in two subsequent microdialysates (Bosch et al., 2016). In the current study, obtaining significant results of two-way ANOVA allowed us to compare all treatment groups to each other at their respective time points and the effect of Ucn3 was not significantly different in comparison to any other treatment group, including control (ACSF) group. In addition, in the previous study Ucn3 was delivered ICV via guide cannula and might have therefore imposed direct inhibitory effect on OT neurons in the hypothalamus. Indeed, excitatory drive onto OT neurons in vole PVN was significantly reduced after application of Ucn3 in hypothalamic slices from voles (Bosch et al., 2016). Ucn3-mediated inhibition of hypothalamic OT neurons has been also demonstrated in rat brain (Chu et al., 2013). Nonetheless, caution needs to be applied when interpreting current Ucn3 results because *ex vivo* experiment indicated that exogenous Ucn3 might potentially interact with the RIA used to measure OT. Thus, if Ucn3 produces false positive results by interacting with the RIA, this might mask its potential inhibitory effect *in vivo*. One-way ANOVA showed an overall significant main effect of Ucn3 treatment on OT content in BNST_dl_ microdialysates but multiple comparisons failed to show differences at any given time point after the drug delivery, which might further support the explanation. Based on these findings, we cannot draw a definitive conclusion about the lack of Ucn3 modulation of OT release in the BNST_dl_. However, despite the technical limitations, exclusion of the Ucn3-treated rats did not affect our main significant findings, including the effects of As2B or CRF application on OT release in the BNST_dl_.

The most significant finding of the current study showed that blockade of CRFR2 by As2B causes a significant increase in OT content in BNST_dl_ microdialysates. This effect is consistent with previously reported results showing an increase in OT release in NaC after As2B was delivered ICV in prairie voles (Bosch et al., 2016). However, in the former study the effect was observed with delay, whereas in the current study it was observed in the first microdialysate after As2B delivery via retrodialysis, suggesting involvement of local CRFR2 in the BNST_dl_. Taken together these results suggest that CRFR2 impose inhibitory effect on OT release in the BNST_dl_, which is unmasked when CRFR2 are blocked, resulting in immediate increase in OT content. Furthermore, the effect of As2B on percentage change of OT content was abolished when combined with CRFR1 antagonist (NBI 35965), suggesting that intact CRFR1 transmission is also required for the increase in OT release.

Like Ucn3, CRF alone did not have any immediate effect on OT release. Therefore, we hypothesized that CRF has a stimulatory effect on OT release via CRFR1, but this is masked by the inhibitory influence of CRFR2. To test this hypothesis, we have applied exogenous CRF while blocking CRFR2 with As2B. Surprisingly, the stimulatory effect of As2B was occluded by application of exogenous CRF. The fact that either the CRFR1 antagonist or CRF blocked the effect of As2B seems puzzling, considering that CRFR1 is a primary receptor for CRF (Hauger et al., 2003a). Since neither Ucn3 nor CRF alone had an immediate effect on OT release, it may be that other members of the CRF peptide family modulate OT release via CRF2R and CRFR1. Ucn2 and Ucn1 are plausible candidates as both Ucn1 and CRF are considered endogenous ligands of CRFR1, whereas Ucn2 and Ucn3 are considered selective CRFR2 ligands (Hauger et al., 2003a). Although Ucn3 is more selective toward CRFR2 than Ucn2 (Ucn2 also binds to the CRF binding protein), the latter has higher affinity toward CRFR2 than Ucn3 (0.25 vs. 14 nM, respectively) (Henckens et al., 2016). Hence, Ucn2 is a plausible mediator of tonic activity of CRFR2 in the BNST_dl_.

Furthermore, the Edinger-Westphal nucleus, which contains neurons producing Ucn1, has been shown to send dense efferent projections to the oval nucleus of the BNST_dl_ (Dos Santos Junior et al., 2015), although another study did not detect Ucn1 immunoreactive processes in rat BNST (Kozicz et al., 1998). Although seemingly contradictory, it is important to note that the latter study used colchicine injections to visualize Ucn1 peptide primarily at the level of cell bodies in the Edinger-Westphal nucleus, which has inevitably prevented Ucn1 immunoreactivity at the level of axons and processes, e.g., in the BNST. Although distinct roles of CRF vs. Ucn1 neurotransmission are not well-understood, differential responses of CRF and Ucn1 systems to acute pain stress have been demonstrated in rat brain (Rouwette et al., 2011). Interestingly, central administration of Ucn1 has been shown to inhibit hyperosmolality-induced vasopressin release in rat SON but CRF demonstrated no effect (Kakiya et al., 1998). In addition, the number of Ucn1 neurons co-expressing FosB in Edinger-Westphal nucleus has been significantly increased in mice genetically predisposed to stress effects and exposed to chronic stress (Farkas et al., 2017). In fact, Ucn1/CRFR2 signaling in the BNST was postulated to play the role in long-term adaptation to and recovery from stress (Kormos and Gaszner, 2013). Strikingly, Ucn1 has higher affinity to CRFR1 than CRF itself (0.17 vs. 1.6 nM, respectively) (Henckens et al., 2016). Furthermore, although it has higher affinity toward CRFR1 than CRFR2 (0.86 nM), it can efficiently bind to both CRFR receptors (Henckens et al., 2016). Thus, Ucn1 rather than CRF might act through CRFR to modulate OT release. All these findings support potentially distinct contributions of CRF vs. Urocortins to the regulation of OT release in the BNST_dl_. Finally, some studies suggested that CRF, Urocortins, and As2B could bind to distinct domains of CRFR2, and therefore affect its transmission differently (Perrin et al., 2003).

As2B also demonstrated a delayed effect on change in OT content, which was affected neither by CRFR1 blockade nor application of CRF. Since the drugs were delivered via retrodialysis, these results suggest that the delayed effect of As2B at 90 min is not mediated by local CRFR in the BNST_dl_ but rather by a feedback mechanism. In fact, As2B-induced immediate increase in OT release (30 min) might activate BNST_dl_ neurons, as OT was shown to excite BNST neurons before (Ingram et al., 1990). From there, PVN-projecting BNST_dl_ neurons that innervate OT cells (Dabrowska et al., 2016) might affect their activity and hence induce delayed terminal OT release in the BNST_dl_. The delayed stimulatory effect of CRF on OT content in BNST_dl_ microdialysates was observed at the same time-point as for As2B alone (90 min), which suggests that both compounds might act via a common mechanism. Although this suggests that the delayed stimulatory effect of CRF on OT release in the BNST_dl_ is primarily mediated by CRFR2, and not CRF1R, high variability observed in the CRF group have inevitably prevented a significant difference, when comparing CRF to CRF co-applied with NBI 35965. Based on current results we propose the following working model of the regulation of OT release in the BNST_dl_. OT release is maintained at a low level by tonic activation of presynaptic CRFR2. Therefore, adding exogenous Ucn3 could not suppress OT release but blockade of CRFR2 immediately increases OT content in BNST_dl_ microdialysates. This effect is also CRFR1-dependent, but not mediated by CRF itself, suggesting the involvement of other members of the CRF peptide family, including Ucn1 acting via CRFR1.

Modulation of OT release most likely also involves intrinsic inhibitory networks as well as extrinsic excitatory innervation of the BNST. CRFR have been shown to display a wide range of modulatory functions in the BNST. CRFR1 activation was shown to potentiate glutamatergic transmission, demonstrated as increased frequency of spontaneous excitatory synaptic currents, suggesting pre-synaptic locus of the CRFR1 action (Kash et al., 2008; Silberman et al., 2013). In contrast, in ventral BNST, CRFR1 activation enhanced postsynaptic responses to GABA, promoting inhibition (Kash and Winder, 2006). Although post-synaptic CRFR2 have been found in posterior BNST (Henckens et al., 2016), our previous electron microscopy study supports pre-synaptic localization of CRFR2 in the BNST_dl_ (Dabrowska et al., 2011). In the CeA, blockade of CRFR2 by As2B facilitated synaptic transmission through presynaptic inhibition of GABA-ergic transmission (disinhibition), whereas blocking CRFR1 had contrasting effect (Fu and Neugebauer, 2008).

CRFR ligands display unique expression patterns throughout the brain but their subcellular locations (e.g., axon vs. dendrite) still remain elusive. In the BNST, the majority of CRFR-labeling (not distinguished between CRFR1 and CRFR2) was observed on the plasma membrane or within dendritic profiles, indicating both post- and/or presynaptic localization (Jaferi and Pickel, 2009). CRFR-labeled axon terminals were shown to form asymmetric, excitatory-type synapses with dendritic spines (Jaferi et al., 2009). In addition, CRFR were also found in dendritic profiles receiving asymmetric, excitatory-type synapses. Presence of the CRFR within some of the excitatory-type terminals that are presynaptic to dendrites expressing CRFR suggests that the CRFR can also affect the activity of local BNST neurons through presynaptic mechanisms (Jaferi et al., 2009). This is consistent with our previous study showing CRFR2-immunoreactive axon terminals and dendrites forming asymmetric, excitatory synapses with dendrites or dendritic spines (Dabrowska et al., 2011).

Therefore, in the BNST, CRFR-immunoreactive axons appear primarily glutamatergic, whereas CRFR-immunoreactive dendrites are primarily GABA-ergic. Only small fraction of CRFR dendrites and terminals in the BNST also contain CRF (Jaferi and Pickel, 2009). As BNST neurons are primarily GABA-ergic (Sun and Cassell, 1993; Dabrowska et al., 2013), glutamatergic CRFR-expressing terminals in the BNST are extrinsic in origin. It is important to note that BNST-projecting hypothalamic OT neurons also express glutamatergic markers (Dabrowska et al., 2011, 2013), suggesting that CRFR2-expressing terminals might co-release both OT and glutamate in the BNST_dl_. Based on these findings, CRFR2 on excitatory OT fibers might also interact with CRFR1 located on dendrites of local GABA-ergic neurons. Hence, the CRF-mediated delayed OT release could be the result of a suppression of local inhibitory networks via CRFR1. On the other hand, glutamate co-released with OT could also increase excitation of local GABA-ergic networks, facilitating long-term effects on OT release.

In addition, both CRFR1 and CRFR2 exert their effects also via changes in intracellular signaling pathways, including extracellular signal-regulated kinase (ERK) and mitogen-activated protein kinase (MAPK), as well as regulation of transcription of downstream target genes. Finally, after CRFR binding, G protein-coupled receptor kinases rapidly phosphorylate the receptors, leading to CRFR internalization, and changes in receptor signaling pathways (Henckens et al., 2016). Delayed changes in gene transcription as well as CRFR trafficking could also lead to the delayed effects of CRFR on OT release.

There are several technical limitations in the current study that need to be addressed. However, extensive statistical tests have been performed to exclude a possibility that these limitations might have interfered with our major findings. For example, slower flow rate during treatment might have resulted in higher OT content in the first microdialysate following drug application. However, we have tested the potential effect the slower rate (for 15 min) might have in 19 control rats infused with ACSF only, and we have not observed any significant effect on OT content. All drugs (including ACSF) were delivered in an identical manner in all treatment groups. In addition, all samples were compared to each other at their respective time points after drug delivery. In fact, As2B is the only group that shows significant increase of OT content in BNST_dl_ microdialysate at 30 min (in comparison to both ACSF and CRF-treated group), whereas CRF-mediated increase has been observed at 90 min after drug administration. Therefore, our major findings that As2B and CRF modulate OT release in the BNST_dl_ are due to their pharmacological effects and are not compromised by the technical limitations. However, it has been demonstrated that microglia and astrocytes express both CRFR1 and CRFR2. If astrocytes and microglia are cellular targets of CRF, gliosis and neuroinflammation around the microdialysis probe might have also affected our results and OT content in BNST_dl_ microdialysates (Stevens et al., 2003).

In addition, our *ex vivo* experiment has shown that Ucn3 might interfere with the RIA used to measure OT content. However, we have re-analyzed the results after exclusion of Ucn3-treated group and it did not affect significant effects of As2B and CRF on OT release. Similarly, even though a probe with a higher cut-off was used for a small group of rats, exclusion of the group from the data analysis also did not change any major findings referenced above.

In order to determine whether the effects of CRFR modulation of OT release are specific to the BNST_dl_, microdialysis samples from rats with off-target cannula placements should also have been analyzed in RIA. Misplacement of the cannulas could be confirmed in only 4 of the 9 excluded rats, the remaining brains showed good cannula trajectory toward the BNST_dl_, but the location of the tip of the probe could not be unequivocally confirmed. Therefore, these samples could not be used as confirmed negative controls.

CRFR ligands used in the study are competitive ligands. As they compete for binding on the same receptor, this might have masked an antagonist effect if an agonist reached the receptor first. However, in regard of co-administration of As2B with CRF, the affinity of As2B for the CRFR2 is very high (1 nM range), whereas the affinity of CRF to CRFR2 is substantially lower (42 nM) (Henckens et al., 2016). Although there still could be differences in diffusion rates between the As2B and CRF from the ACSF to extracellular space, this is unlikely as they are used at the same concentration (10 μM) and both peptides have similar molecular weights (4,757.52 and 4,041.69 g/mol, CRF and As2B, respectively). As As2B has much higher affinity and a smaller molecular size, it would presumably arrive at CRFR2 site faster and bind more efficiently than the agonist, CRF. In regard to co-application of CRF and NBI 35965, the latter is a non-peptide CRFR1 antagonist and therefore has a considerably smaller molecular weight of 437.79 grams/mol. Both CRF and NBI 35965 were used in the same concentration of 10 μM but because of the smaller size, NBI 35965 would presumably diffuse and reach CRFR1 site faster than CRF. However, caution needs to be applied as although NBI 35965 has much higher affinity to CRFR1 (4 nM range) vs. CRFR2 (>10,000 nM), it has a lower affinity than a CRF for CRFR1 (1.6 nM) (Henckens et al., 2016). Co-application of NBI 35965 and As2B should not be an issue, because these are selective antagonists of CRFR1 and CRFR2, respectively.

Stress activation of the HPA axis and the anxiogenic and depressive-like effects of CRF are primarily mediated by activation of CRFR1 (Sahuque et al., 2006; Kehne and Cain, 2010), (but see Coste et al., 2000). However, there are many conflicting findings on the behavioral effects of CRFR2 activation (Valdez et al., 2003; Risbrough et al., 2004), gene deletion (Coste et al., 2000; Kishimoto et al., 2000), or blockade (Takahashi et al., 2001). In fact, growing evidence suggests that the role of CRF peptide family members in stress and anxiety is largely brain-region specific (Janssen and Kozicz, 2013; Henckens et al., 2016). For example, lack of CRFR1 on glutamatergic neurons in the amygdala reduces anxiety, whereas selective deletion of CRFR1 in midbrain dopaminergic neurons increases anxiety-like behavior (Refojo et al., 2011). The behavioral effects of CRFR2 in the BNST have also been inconsistent (Sahuque et al., 2006; Lebow et al., 2012; Elharrar et al., 2013). In addition, the BNST is a heterogeneous structure containing sub-nuclei that express variety of neuropeptides, and activation of different sub-nuclei often leads to contrasting physiological and behavioral effects (Daniel and Rainnie, 2016). Therefore, the role of CRFR1 and CRFR2 should also be investigated in BNST sub-nuclei dependent manner. Based on our previous (Dabrowska et al., 2011) and current findings we propose that CRF receptors in the BNST_dl_ may function to modulate stress and anxiety-like behavior via an interaction with the OT system. We have recently shown that OT neurotransmission in the BNST_dl_ facilitates cued fear learning (Moaddab and Dabrowska, 2017). Therefore, CRFR might modulate fear and anxiety responses at least partially via modulation of OT release in the BNST_dl_. Our current findings imply that the interaction between CRF receptors and OT release might affect a whole array of responses, including sensitivity to stressors, fear and anxiety, as well as depressive-like behaviors (Coste et al., 2000; Kishimoto et al., 2000). Future studies will focus on investigating the role of CRF-peptide family members in stress-induced OT release in the BNST_dl_.

## Author contributions

JD designed the study; DM acquired data; DM and JD analyzed and interpreted data, and wrote the manuscript.

### Conflict of interest statement

The authors declare that the research was conducted in the absence of any commercial or financial relationships that could be construed as a potential conflict of interest.
